# Attitudes towards the Potential Use of Aversive Geofencing Devices to Manage Wild Elephant Movement

**DOI:** 10.3390/ani13162657

**Published:** 2023-08-18

**Authors:** Surendranie J. Cabral de Mel, Saman Seneweera, Ashoka Dangolla, Devaka K. Weerakoon, Tek Maraseni, Benjamin L. Allen

**Affiliations:** 1 Institute for Life Sciences and the Environment, University of Southern Queensland, Toowoomba, QLD 4350, Australia; surendranie.cabral@gmail.com (S.J.C.d.M.); tek.maraseni@usq.edu.au (T.M.); 2 National Institute of Fundamental Studies, Kandy 20000, Sri Lanka; seneweera@gmail.com; 3 School of Agriculture and Food, Faculty of Sciences, The University of Melbourne, Parkville, VIC 3010, Australia; 4 Department of Veterinary Clinical Sciences, University of Peradeniya, Peradeniya 20400, Sri Lanka; adangolla@gmail.com; 5 Department of Zoology and Environmental Sciences, University of Colombo, Colombo 00300, Sri Lanka; devakaw@gmail.com; 6 Northwest Institute of Eco-Environment and Resources, Chinese Academy of Sciences, Lanzhou 730000, China; 7 Centre for African Conservation Ecology, Nelson Mandela University, Port Elizabeth 6034, South Africa

**Keywords:** conservation, electric shock collars, *Elephas maximus*, human-elephant conflict, public opinion, virtual fencing, wildlife management

## Abstract

**Simple Summary:**

Human-elephant conflict (HEC) has intensified in the recent decades and poses a great threat to Asian elephant conservation. Aversive geofencing devices (AGDs) or animal-borne satellite-linked shock collars might become a useful tool to help reduce HEC incidents. AGDs may be used on problem causing elephants, to train them to move away from human-dominated landscapes by associating the receipt of electric shocks with preceding audio warnings given from the AGD as they approach virtual boundaries. We assessed the opinions of experts, farmers, and others who have and have not experienced HEC towards the potential use of AGDs on Asian elephants. Most respondents had positive opinions on the potential effectiveness of AGDs in managing elephant movement (62.2%). About 62.8% respondents also expressed positive responses for the acceptability of AGDs if pilot studies with captive elephants have been successful in managing their movements. Some respondents perceived AGDs to be unacceptable because they are unethical or harmful and would be unsuccessful given wild elephants may respond differently to AGDs than captive elephants. Respondents identified several potential challenges for implementing AGDs as an elephant management tool. These issues need attention when developing AGDs to increase support from stakeholders and to effectively reduce HEC incidents in the future.

**Abstract:**

Aversive geofencing devices (AGDs) or animal-borne satellite-linked shock collars might become a useful tool to mitigate human-elephant conflict (HEC). AGDs have the potential to condition problem elephants to avoid human-dominated landscapes by associating mild electric shocks with preceding audio warnings given as they approach virtual boundaries. We assessed the opinions of different stakeholders (experts, farmers, and others who have and have not experienced HEC; n = 611) on the potential use of AGDs on Asian elephants. Most respondents expressed positive opinions on the potential effectiveness of AGDs in managing elephant movement (62.2%). About 62.8% respondents also provided positive responses for the acceptability of AGDs if pilot studies with captive elephants have been successful in managing their movements. Some respondents perceived AGDs to be unacceptable because they are unethical or harmful and would be unsuccessful given wild elephants may respond differently to AGDs than captive elephants. Respondents identified acceptability, support and awareness of stakeholders, safety and wellbeing of elephants, logistical difficulties, durability and reliable functionality of AGDs, and uncertainties in elephants’ responses to AGDs as potential challenges for implementing AGDs. These issues need attention when developing AGDs to increase support from stakeholders and to effectively reduce HEC incidents in the future.

## 1. Introduction

The majority of Asian elephant *Elephas maximus* populations inhabit fragmented habitats dispersed among human-dominated landscapes. Thus, negative interactions between humans and elephants are inevitable [1,2,3,4,5]. Human-elephant conflict (HEC) is spread across all the 13 Asian elephant range countries (Bangladesh, Bhutan, Cambodia, China, India, Indonesia, Laos, Malaysia, Myanmar, Nepal, Sri Lanka, Thailand, and Vietnam) and is the biggest challenge for the conservation of this endangered species [6]. As a result of HEC, hundreds of human lives are lost each year and farmers in rural communities experience large scale economic losses from crop and property damage [7,8,9,10,11]. Hundreds of elephants also die annually from intentional or unintentional human actions that directly or indirectly result from HEC. Unintentional deaths of elephants may be caused by accidents such as falling into agricultural wells and abandoned quarries or gem pits, colliding with trains, traps or snares setup for other animals, and electrocution from contact with low lying electric power lines or lethal electric fences [5,12,13,14,15,16]. Intentional deaths occur from poaching for elephant body parts [17,18,19], but the majority are due to retaliation against conflict-causing elephants by using jaw bombs (explosives placed in fruits which are then offered to elephants), or poisoning or shooting [15,19,20]. Despite the religious and cultural significance of elephants in the region [21,22,23], many people may be driven to retaliate out of desperation to protect their lives and livelihoods.

Lethal control is considered by most people to be unacceptable [24] and is no longer permitted in most range countries [25,26]. Rather, removal of problem elephants from conflict areas by translocation, domestication, or driving them into protected areas are commonly practiced to mitigate HEC [27,28,29,30,31]. Government authorities are compelled to take such extreme action due to public and political pressure. However, these measures have typically proven to be ineffective in reducing HEC and rather intensify it or severely compromise the wellbeing of elephants, sometimes even causing death to the animal [27,28,32,33,34,35]. HEC mitigation approaches readily available to people, such as various physical and biological barrier methods or repellent techniques, have numerous drawbacks or are ineffective in the long-term given that elephants quickly habituate to them [36,37,38]. Electric fences may be the most effective HEC mitigation method, if properly built and maintained [39,40,41]. But inherent problems of electric fences, such as lack of flexibility once they are constructed, restriction of movement, and access to resources for both elephants and non-targeted species [25,42,43] emphasise the need to explore more innovative methods, to provide effective solutions to mitigate HEC.

Animal-borne satellite-linked electric shock collars or aversive geofencing devices (AGDs) are a potentially effective but perhaps controversial tool suggested for managing conflicts with Asian elephants [26,38]. AGDs were first used as a virtual fencing system for domestic pets and were designed to deliver a shock when an animal wearing the collar approached a signal-emitting wire hidden around a predetermined area [44]. Modern AGDs, now commercially used on livestock species have real-time Global Positioning System (GPS) tracking capabilities and can be programmed to deliver an audio warning followed by a mild electric shock automatically whenever an animal attempts to cross a virtual boundary [45]. These devices have successfully trained cattle *Bos taurus* and sheep *Ovis aries* to associate an audio warning with an unpleasant or aversive electric shock and avoid it by altering their movement whenever they hear the audio warning [46,47,48]. Similarly, AGDs may have the potential to condition elephants to alter their movement and avoid human-dominated landscapes. This approach has also been tested on several wild canid species to minimise human-wildlife conflict or prevent predation, and has been identified as an acceptable alternative to lethal control [49,50,51,52]. AGDs could also act as an early warning system which can automatically send a message to mobile phones warning villagers and wildlife managers about the presence of a problem elephant near the village and the potential breaches of virtual fences by those elephants that ignore the audio warnings and electric shocks [38]. AGDs could, therefore, help reduce conflict incidents with problem causing elephants.

Although the use of electric shocks to manage domestic pets and livestock species have been in existence for many decades [53], their use is still debated over animal ethics and welfare concerns [54,55,56,57,58]. Part of the reason for this debate may be the many nuances associated with the way electric shocks are used with these species, e.g., the strength of the shock, or whether shocks are delivered by humans or if animals can avoid the shock if they choose, and the possible stress that it would cause on the animal. Non-lethal electric fences used for elephants typically deliver electric shocks of 5500–10,000 V with very low amperage (~5 mA) and a pulse duration of about a few milliseconds [25,40,59,60], and are generally perceived as an acceptable HEC mitigation tool [61]. AGDs used on livestock are also designed to deliver shocks with similar characteristics, but use a much lower voltage (e.g., ~800 V [47,62]) and lower energy than that given from electric fences used for these species [63]. Similarly, a much milder electric shock than that used in elephant electric fences might be used with AGDs for elephants as well [64]. However, compared to capturing and attaching collars with AGDs on other smaller or domesticated animals, fitting AGDs on elephants would pose a risk to both elephants and humans involved in the collaring process [65].

Stakeholders’ interests and ideas about managing wildlife, especially on controversial management tools may differ [66,67,68,69,70,71,72]. Public opinion can also be stronger when it comes to large, charismatic, and symbolic species [73,74,75]. Understanding the opinions of stakeholders towards potentially controversial but otherwise effective human-wildlife conflict mitigation approaches is important for their successful implementation. We previously conducted pilot studies on AGDs which revealed that electric shocks (~4000 V, ~51.7 µs, with no resistance) from a modified dog-training collar delivered on the neck of captive Asian elephants produces desirable aversive responses, and that there is potential to condition elephants to avoid the shock with a prior audio warning [64]. Furthermore, our assessment of behavioural and physiological stress responses to electric shocks of these captive elephants showed that the expected increase in acute stress responses returned to normal levels soon afterwards [76]. These studies revealed promising results for the potential use of AGDs to manage wild elephants, but their successful use may depend on their acceptability just as much as their efficacy [36,77], which was not assessed in these studies. Evaluating and considering the views of experts and non-experts, and those who are and are not affected by HEC is important for developing consensus around the successful deployment of AGDs. Therefore, in this study we aimed to assess the perceptions of different stakeholders towards the potential use of AGDs as an HEC mitigation tool. We further explored the respondents’ stated reasons for unacceptability and solicited their views on potential challenges that could be faced when developing AGDs. The overall aim of the study was to identify issues that may need further research as the development and use of AGDs continue.

## 2. Materials and Methods

### 2.1. Survey Administration

We conducted an online and paper-based questionnaire survey from May–October 2022 to evaluate people’s opinion on the potential use of AGDs as an HEC mitigation tool. Participants were enlisted using convenience and snowball sampling. The online survey targeted citizens or residents of the Asian elephant range countries, as well as experts from around the world involved in research or other work related to Asian elephants. The online survey was created using the University of Southern Queensland web-based survey tool and was shared with potential participants using social media. Email addresses of experts were obtained from published research articles or relevant organisation websites, and personal emails were sent with the survey link inviting them to participate in the survey. The paper-based survey was conducted in Sri Lanka, a country experiencing very high levels of HEC incidents [8], and targeted the rural farming communities in areas experiencing HEC with limited facilities to participate in the online survey. With the support of volunteer field assistants, self-administered survey forms were distributed among people and completed forms were collected later. The survey was made available in English as well as Sinhala and Tamil languages, the two main languages spoken in Sri Lanka. Individual respondents were not identifiable from the data (i.e., individual identifiers were not collected) and all respondents provided implied consent by completing and submitting the survey voluntarily. This study was approved by the Human Research Ethics Committee of the University of Southern Queensland, Australia (H21REA209) and the Institute of Biology, Sri Lanka (ERC IOBSL 258 01 2022).

### 2.2. Survey Questions

**Figure 1:**
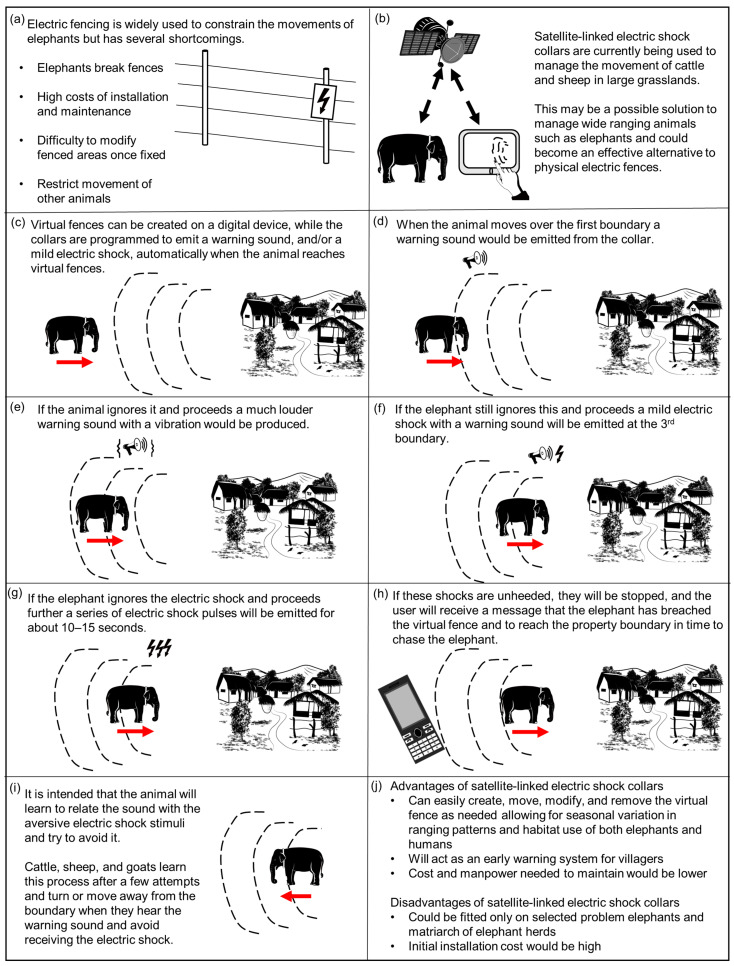
How aversive geofencing devices (satellite-linked electric shock collars) could be used to manage elephant movement.

The concept of AGDs was briefly explained with illustrations at the beginning of the survey to provide respondents with a basic understanding on how AGDs are expected to manage elephant movement (Figure 1). The initial section of the survey collected demographic information of respondents such as age, gender, education level, citizenship, religion, and involvement in agriculture and in work related to Asian elephants (Table S1). Respondents’ experiences with HEC were collected by asking the severity level and type of HEC they faced (Table S2). People’s opinions on AGDs were collected using four closed-ended questions and three optional open-ended questions. The four closed-ended questions were 5-point Likert-type questions with responses on a bipolar scale (−2 to +2). These questions considered (1) How likely it would be for elephants to learn to avoid an electric shock from AGDs, (2) How acceptable it is to give an electric shock to an elephant using an AGD, (3) How effective AGDs would be in managing elephant movement, and (4) Would the use of AGDs on wild elephants be acceptable if pilot studies conducted with captive elephants are proven successful. The three optional open-ended questions collected respondents’ feedback on (1) Reasons for unacceptability, (2) Potential challenges in implementing AGDs, and (3) Any other comments on the use of AGDs.

### 2.3. Data Analysis

We analysed responses from 611 respondents based on three social groups (experts, farmers and others) and whether or not they have experienced HEC (HEC or no HEC). Respondents were categorised as a “farmer” or “expert” if they had indicated their involvement in farming (annual crops or perennial crops or livestock) or work related to Asian elephants in their responses to the survey (see Table S1). Those who did not belong to either of the groups were categorised as “other”. Respondents were categorised as “HEC” if they had selected a severity level of HEC they experienced and/or mentioned at least one HEC related problem they have experienced (see Table S2), while the remaining respondents were categorised as “no-HEC”.

We analysed the responses for the Likert-type questions using a logistic-regression model (a generalised linear model with a binomial distribution and a logit link function), by collapsing the responses to a binary variable (−2, −1 and 0 as a “negative/neutral” response and +1, +2 as a “positive” response). Such transformation of scale avoids issues with low frequency of responses in some response categories and simplifies the interpretation of data [78,79]. We used the ‘glm’ function and the forestplot package [80] in the R statistical software [81] for this analysis. We used the Potential for Conflict Index_2_ (PCI_2_) [82] to examine the mean responses given on the five-point scale (−2 to +2) and the level of consensus within the six groups: expert-HEC, expert-no HEC, farmer-HEC, farmer-no HEC, other-HEC, and other-no HEC. PCI_2_ values range between 0 and 1 and depict the dispersion within the sample, with 0 indicating highest consensus within a group of respondents and 1 indicating the lowest consensus within a group (i.e., all responses are divided between the extreme response categories equally). We used the programs provided by Vaske et al. [82] to calculate and graph PCI_2_ and mean values. The centre and the size of the bubble in the graph depict the mean score on the scale of the y axis and the PCI_2_ value, respectively. High potential for conflict within a group is depicted by larger bubbles and low potential for conflict within a group is depicted by smaller bubbles. Each analysed question (item) was reduced to shorter phrases for the convenience of display and are italicised when mentioned in the Results section (below). Table S3 contains the full-length questions. Responses to the open-ended questions were categorised according to themes, and due to the ambiguity in interpretation of the responses the approximate number of respondents commenting under each category are given within parenthesis.

## 3. Results

Out of the 611 responses we analysed in this study, 25.9% (n = 158) were classified as experts. Of these, 65 had experienced HEC and 93 had not. These experts included 70 Sri Lankan citizens, 60 from other range countries, and 28 from non-range countries (Table S1). Farmers comprised 18.3% (n = 112) of the respondents. Of these, 85 had experienced HEC and 27 had not. The remaining 341 respondents classified as others included 83 who had experienced HEC and 258 who had not. A total of 38.1% (n = 233) of respondents had experienced HEC (Table S2).

**Figure 2:**
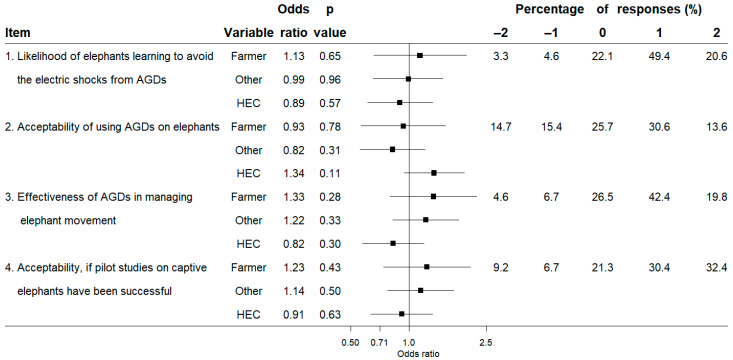
Forest plot for the logistic regression on the attitudes towards use of aversive geofencing devices (AGDs) as a human-elephant conflict (HEC) mitigation tool by farmers and others relative to experts and those who have experienced HEC relative to those who have not along with overall percentage responses for each item. Black squares and horizontal lines indicate the odds ratio and the 95% confidence interval, respectively.

**Figure 3:**
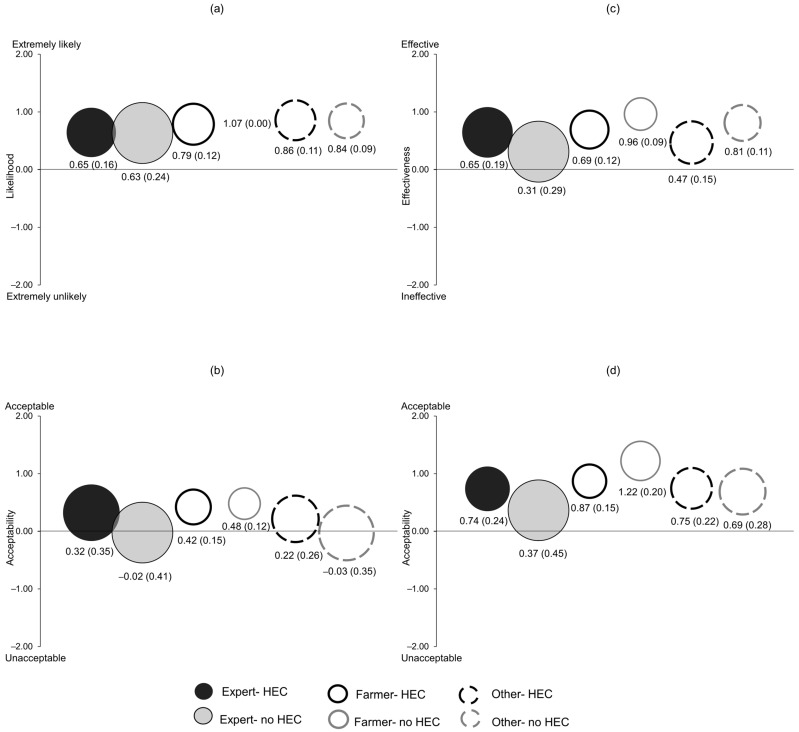
Bubble graphs for mean and Potential for Conflict Index_2_ (PCI_2_) on the perception of potential use of aversive geofencing devices (AGDs) as a human-elephant conflict (HEC) mitigation tool among experts, farmers and others who have and have not experienced HEC. (**a**) Likelihood of elephants learning to avoid the electric shocks from AGDs, (**b**) Acceptability of using AGDs on elephants, (**c**) Effectiveness of AGDs in managing elephant movement, (**d**) Acceptability, if pilot studies on captive elephants have been successful. Centre of the bubble indicates the mean score (on the scale of the y axis) and bubble size illustrates the magnitude of PCI_2_, with larger bubbles indicating low consensus among respondents within groups. Values under each bubble indicate mean and PCI_2_ value within parenthesis.

Overall, more than 50% of all respondents had positive responses for all items except for *acceptability of using AGDs on elephants*, for which only 44.2% (n = 270) of respondents had positive opinions (Figure 2). The logistic regression did not reveal detectable differences in opinions for all four items between the stakeholder groups; farmers and others compared to experts or those who had experienced HEC compared to those who had not (*p* > 0.05, Figure 2). All respondent groups had positive mean scores for *likelihood of elephants learning to avoid the electric shocks from AGDs* and e*ffectiveness of AGDs in managing elephant movement* with relatively high consensus (PCI_2_ range = 0.00–0.20, Figure 3). Mean scores for *acceptability of using AGDs on elephants* ranged from −0.03 to 0.48 (PCI_2_ range = 0.12–0.41), while the mean scores for *acceptability, if pilot studies on captive elephants have been successful* ranged from 0.37 to 1.22 (PCI_2_ range = 0.15–0.45, Figure 3). Provided with the condition ‘if pilot studies on captive elephants have been successful’, acceptability scores increased by a mean difference of 0.57 (t = 11.50, df = 610, *p* < 0.001).

Of the total respondents, 15.9% (n = 97) selected “unacceptable” or “somewhat unacceptable” for *acceptability, if pilot studies on captive elephants have been successful* (Figure 2). These respondents were represented by all six groups: 13.8% of expert-HEC (n = 9), 30.1% of expert-no HEC (n = 28), 7.1% farmer-HEC (n = 6), 11.1% of farmer-no HEC (n = 3), 14.5% of other-HEC (n = 12), and 15.1% of other-no HEC (n = 39) (Table S3). Of these 97 respondents, 61 offered reasons for the unacceptability of AGDs, which mostly indicated that they perceived AGDs to be unethical, cruel or harmful to elephants (~41), and that it is an approach they considered to be unfeasible or would be unsuccessful because wild elephant behaviour would be different from captive elephants (~25). About 300 respondents provided feedback on the potential challenges in implementing AGDs and/or other comments. These comments can be categorised under five themes: (1) acceptability, support and awareness of stakeholders (~46), (2) safety and wellbeing of elephants (~68), (3) logistical difficulties (~169), (4) durability and reliable functionality of AGDs (~91), and (5) uncertainties in elephants’ responses to AGDs (~62). Selected comments offered as reasons for unacceptability and potential challenges are discussed below and provided as Supplementary Material (Tables S4 and S5, respectively).

## 4. Discussion

We explored the perceptions towards the potential use of AGDs to manage elephant movement by surveying different stakeholder groups whose opinions and support is vital for the successful implementation of AGDs as an HEC mitigation tool. We found that respondents had similar views towards AGDs regardless of whether they were experts, farmers or others, and whether or not they had personal experience with HEC; each group largely felt the same towards AGDs (Figure 2). Elephants are intelligent animals with superior cognitive skills [83,84], a trait acknowledged by the general public with usage of phrases such as “memory like an elephant” or “an elephant never forgets” [85,86]. This understanding may have contributed to all respondent groups agreeing on the likelihood of elephants learning to associate the AGDs’ warning sound with the impending electric shock, thereby avoiding the shock and highlighting the potential effectiveness of AGDs in managing elephant movement (Figure 3a,c). When questioned about the acceptability of managing elephants in this way, the expert-no HEC and other-no HEC groups were relatively neutral, while all other groups considered it somewhat acceptable (Figure 3b). People tend to perceive a novel HEC mitigation tool as increasingly favourable as their knowledge on its effectiveness improves [77]. Similarly, our results suggested that if evidence can be provided that AGDs can effectively manage the movement of captive elephants, then the acceptability of using AGDs on wild elephants would increase among all groups (Figure 3d). However, the relatively lower acceptability and higher potential for conflict within the expert-no HEC group, even if such evidence is provided, indicates that building consensus among all experts on AGDs may pose some challenges.

Most people were either ambivalent or considered AGDs to be acceptable (Figure 1), but those who considered AGDs to be unacceptable may be categorised into two main groups based on their stated reasons for unacceptability: (1) those who see AGDs as unethical or harmful, and (2) those who feel that AGDs will be unsuccessful in managing wild elephant movements (Table S4). These opinions may be due to “conflict over values and conflict over evidence”, as highlighted by Donfrancesco et al. [72]. If scientific evidence can be provided on the effectiveness of AGDs from preliminary trials with wild elephants, it will help develop consensus with the latter group. But the views of those who consider AGDs to be unethical might vary under different severity levels of HEC [87], for example, when HEC results in frequent death of humans and elephants, rather than a low frequency of crop damage. Obtaining social acceptability and the support of stakeholders is important and was also suggested by our respondents as a potential challenge for implementing AGDs. One respondent even pointed out that some may consider elephants as non-human persons with rights (Table S5, see also Riddle, [88]; Lev and Barkai, [89] and Locke, [90]), and therefore, using methods where humans ‘control’ animals may be perceived as unethical. People’s opinion towards management approaches may change with more awareness of the actual situation [74]. Proper dialog between relevant groups on the severity of HEC experienced in many regions and whether retaliatory killing, other HEC mitigation approaches or use of AGDs would be ethically justifiable and effective in such situations will be important.

Respondents questioned the impact on the mental and physical wellbeing of elephants in response to electric shocks. Previous studies conducted on other species [63,91,92,93,94,95] and with captive Asian elephants [76] showed that the expected increase in acute stress levels measured using behavioural (e.g., aversive or anxiety/stress related behaviours) and physiological (e.g., cortisol hormone levels, heart rate, body temperature) responses to electric shock rapidly returned to baseline levels soon after experiencing them. Further, by ensuring that stimuli are delivered only when the elephant moved in the ‘wrong’ direction and not based on its location, will permit elephants to learn accurately and move in the ‘right’ direction to avoid the shock [96]. Coupling the electric shock with the audio warning provides the ability for elephants to predict and control the receipt of shocks [97,98], which would further reduce their acute stress response levels as shown for cattle [94]. After elephants learn to predict and control the receipt of shock, chronic stress responses to AGDs might be negligible compared to baseline levels [63,95]. The impact of electric shocks from AGDs on elephants’ wellbeing would, therefore, be negligible or no different to that experienced with other HEC mitigation tools, though this needs further investigation.

Many respondents had reasonable concerns about logistical challenges during implementation of AGDs. For example, collaring wild elephants and planning virtual fences will be a very difficult task (Table S5, see also Pastorini et al. [65]). Fitting collars on wild elephants to monitor their movements has been practiced for many years [4,17,28,32,65], but AGDs should preferably be used on selected problem elephants or in HEC scenarios where no other acceptable approach has been effective [38]. AGDs should not be considered as a replacement for all other existing HEC mitigation approaches. While elephant herds are sometimes known to raid crops [32,99,100], it is primarily the male elephants that are involved in direct confrontations with people [32,101] and crop raiding [102,103,104,105]. AGDs could help reduce these HEC incidents if used on those types of problem elephants. To successfully reduce HEC incidents with problem elephants using AGDs, it is important to incorporate both human and elephant needs and ensure connectivity between elephant habitats when planning virtual fences [106,107]. Therefore, conducting baseline studies to understand the land use patterns of humans and elephants and the unique situation of HEC where AGDs are to be implemented are required to design virtual fences appropriately [38].

Our study also revealed several other potential challenges that should be resolved during the research and development process of AGDs. These include ensuring reliability of the technology (e.g., consistency and accuracy of delivering audio and electrical stimuli, maintaining uninterrupted satellite communication), durability of the device (e.g., weather resistance, long lasting battery life), and resolving uncertainties in elephant’s behavioural responses to electric shocks (e.g., individual variations in responses or potential to show aggressive behaviours, possibility to learn through social facilitation and possibility of habituation to the stimuli) (Table S5). These possible challenges have been identified and discussed in Cabral de Mel et al. [38,64] and should be further investigated. Given the general support or lack of widespread opposition towards AGDs, the next step would be designing and developing a wearable, prototype AGD and optimising it by further testing with captive elephants to provide evidence on its efficacy in managing elephant movement with minimum impact on elephant wellbeing. Once many of the uncertainties are resolved and with the acceptance and support of stakeholders, this prototype device can then be modified (if necessary) and trialled on selected wild elephants in a high-HEC area to further assess its efficacy.

## 5. Conclusions

AGDs are an innovative tool used to manage animal movement and could help manage Asian elephant movement too. However, the successful use of AGDs is most likely to occur when consideration is given to all stakeholder views. Our study showed that the majority of respondents had confidence that elephants will learn to avoid the shock from AGDs by altering their movement when presented with the audio warning and thereby effectively mitigate HEC. Most respondents were also neutral or generally accepting towards the use of AGDs. Providing evidence that AGDs reliably and effectively manage captive elephant movement without compromising elephant wellbeing would further improve the acceptability of AGDs among stakeholders. Such favourable views, especially among people experiencing HEC, are important to receive support for the successful implementation of AGDs. We expect that there will still be a small proportion of stakeholders who will object to the use of AGDs on elephants irrespective of its efficacy or general acceptability in managing captive elephant movement, so such views should be further evaluated to discover how they may vary depending on different HEC scenarios. Proper communication and awareness among stakeholders about the outcome of preliminary research on AGDs should build consensus among stakeholders around the more widespread use of AGDs in the future.

## Data Availability

The authors confirm that the supporting data of these findings are available within the article and its Supplementary Materials. The summarised data generated during the current study are available from the corresponding author on reasonable request.

## Supplementary Materials

The following supporting information can be downloaded at: https://www.mdpi.com/article/10.3390/ani13162657/s1, Table S1: Socioeconomic details of all respondents analysed in the study; Table S2: Personal experience of respondents in human-elephant conflict (HEC); Table S3: Full length question of the items analysed in the study; Table S4: Selected list of reasons provided by respondents for unacceptability of using aversive geofencing devices on elephants; Table S5: Selected list of feedback received from respondents for potential challenges and other comments on aversive geofencing devices.
