# Supplementary material for: Attitudes towards the Potential Use of Aversive Geofencing Devices to Manage Wild Elephant Movement
Source: Animals (Basel). 2023 Aug 18;13(16):2657. doi: 10.3390/ani13162657 (PMC10451760; doi:10.3390/ani13162657)
Supplement: Supplementary file 1 [file animals-13-02657-s001.zip › animals-2548808-supplementary.pdf]

Supplementary information for 'Attitudes towards the potential use of aversive geofencing devices to manage wild elephant movement'.

Table S1. Socioeconomic details of all respondents analysed in the study

		Expert-HEC[1] (n=65)		Expert- no HEC[1] (n=93)		Farmer-HEC[1] (n=85)		Farmer- no HEC[1] (n=27)		Other-HEC[1] (n=83)		Other- no HEC[1] (n=258)		Total (n=611)	
Item	Categories	n	%	n	%	n	%	n	%	n	%	n	%	n	%
Age	18-35	21	32.3	37	39.8	33	38.8	17	63.0	56	67.5	159	61.6	323	52.9
	36-55	35	53.8	38	40.9	31	36.5	7	25.9	20	24.1	66	25.6	197	32.2
	>56	9	13.8	18	19.4	21	24.7	3	11.1	7	8.4	33	12.8	91	14.9
Gender	Female	16	24.6	35	37.6	23	27.1	7	25.9	46	55.4	161	62.4	288	47.1
	Male	49	75.4	58	62.4	62	72.9	20	74.1	37	44.6	97	37.6	323	52.9
Highest education qualification	Primary	0	0.0	0	0.0	3	3.5	0	0.0	0	0.0	3	1.2	6	1.0
	Secondary	4	6.2	5	5.4	18	21.2	6	22.2	9	10.8	27	10.5	69	11.3
	Tertiary	61	93.8	88	94.6	64	75.3	21	77.8	74	89.2	228	88.4	536	87.7
Religion	Buddhism	26	40.0	28	30.1	77	90.6	21	77.8	58	69.9	155	60.1	365	59.7
	Christianity	5	7.7	15	16.1	1	1.2	4	14.8	8	9.6	58	22.5	91	14.9
	Hinduism	18	27.7	12	12.9	6	7.1	1	3.7	10	12.0	19	7.4	66	10.8
	Islam	3	4.6	6	6.5	0	0.0	0	0.0	3	3.6	7	2.7	19	3.1
	Other	12	18.5	26	28.0	1	1.2	1	3.7	4	4.8	15	5.8	59	9.7
	Not applicable	1	1.5	6	6.5	0	0.0	0	0.0	0	0.0	4	1.6	11	1.8
Citizenship	Bangladesh	1	1.5	3	3.2	0	0.0	0	0.0	0	0.0	1	0.4	5	0.8
	China	0	0.0	2	2.2	0	0.0	0	0.0	0	0.0	0	0.0	2	0.3
	India	12	18.5	17	18.3	1	1.2	1	3.7	2	2.4	15	5.8	48	7.9
	Indonesia	0	0.0	1	1.1	0	0.0	0	0.0	0	0.0	0	0.0	1	0.2
	Malaysia	0	0.0	3	3.2	0	0.0	0	0.0	0	0.0	0	0.0	3	0.5
	Myanmar	1	1.5	3	3.2	0	0.0	0	0.0	0	0.0	1	0.4	5	0.8
	Nepal	7	10.8	1	1.1	0	0.0	0	0.0	1	1.2	1	0.4	10	1.6
	Sri Lanka	31	47.7	39	41.9	84	98.8	26	96.3	80	96.4	239	92.6	499	81.7
	Thailand	5	7.7	1	1.1	0	0.0	0	0.0	0	0.0	1	0.4	7	1.1
	Vietnam	3	4.6	0	0.0	0	0.0	0	0.0	0	0.0	0	0.0	3	0.5
	Other	5	7.7	23	24.7	0	0.0	0	0.0	0	0.0	0	0.0	28	4.6

Item	Categories	Expert-HEC[1] (n=65)		Expert- no HEC[1] (n=93)		Farmer-HEC[1] (n=85)		Farmer- no HEC[1] (n=27)		Other-HEC[1] (n=83)		Other- no HEC[1] (n=258)		Total (n=611)	
		n	%	n	%	n	%	n	%	n	%	n	%	n	%
Involvement in agriculture	Farmer-annual crops					71	83.5	14	51.9					85	13.9
	Farmer-perennial crops					9	10.6	12	44.4					21	3.4
	Farmer-livestock					5	5.9	1	3.7					6	1.0
	Government officer-Agriculture	7	10.8	0	0.0					13	15.7	3	1.2	23	3.8
	Researcher or educator-Agriculture	18	27.7	19	20.4					4	4.8	19	7.4	60	9.8
	General interest in Agriculture	10	15.4	14	15.1					29	34.9	70	27.1	123	20.1
	Not applicable	30	46.2	60	64.5					37	44.6	166	64.3	293	48.0
Involvement in work related to Asian elephants	Member of the IUCN[2] Asian elephant specialist group	11	16.9	20	21.5									31	5.1
	Zoo based organisation housing Asian elephants	8	12.3	11	11.8									19	3.1
	Non-governmental organisation working on Asian elephants	22	33.8	43	46.2									65	10.6
	Research/ educator on Asian elephants	38	58.5	64	68.8									102	16.7
	Government organisation working on Asian elephants	19	29.2	8	8.6									27	4.4

[1] HEC: human-elephant conflict

[2] IUCN: International Union for Conservation of Nature

Table S2. Personal experience of respondents in human-elephant conflict (HEC)

Item	Categories	Expert- HEC (n=65) n	%	Farmer- HEC (n=93) n	%	Other- HEC (n=83) n	%	Total (n=233) n	%
Severity of HEC	No problem	14	21.5	2	2.4	21	25.3	37	15.9
	Minor problem	15	23.1	15	17.6	27	32.5	57	24.5
	Moderate problem	15	23.1	32	37.6	25	30.1	72	30.9
	Major problem	12	18.5	23	27.1	8	9.6	43	18.5
	Serious problem	9	13.8	13	15.3	2	2.4	24	10.3
Types of problems experiences	Property damage	29	44.6	25	29.4	21	25.3	75	32.2
	Crop damage	38	58.5	77	90.6	52	62.7	167	71.7
	Physical injury	19	29.2	4	4.7	8	9.6	31	13.3
	Death of family member	8	12.3	7	8.2	5	6.0	20	8.6
	Fear	27	41.5	25	29.4	30	36.1	82	35.2
	Damage to livestock	7	10.8	6	7.1	7	8.4	20	8.6

Table S3. Full length question of the items analysed in the study

Question	Item	Responses	Percentage of responses (%) Expert-HEC[1] (n=65)	Expert-no HEC[1] (n=93)	Farmer-HEC[1] (n=85)	Farmer-no HEC[1] (n=27)	Other-HEC[1] (n=83)	Other-no HEC[1] (n=258)
1. Satellite linked shock collars may have the potential to manage elephant movement by acting as virtual fences. How likely do you think elephants would be able to learn to relate the warning sound with the electric shock and avoid the shock like cattle and sheep?	Likelihood for elephants to learn to avoid the electric shocks from AGDs[2]	Extremely unlikely	4.6	6.5	2.4	0.0	2.4	2.7
		Unlikely	6.2	9.7	5.9	0.0	4.8	2.3
		Neutral	18.5	15.1	23.5	18.5	24.1	24.8
		Likely	61.5	51.6	47.1	55.6	42.2	48.1
		Extremely likely	9.2	17.2	21.2	25.9	26.5	22.1
2. How acceptable it is to give an electric shock to an elephant using a GPS collar	Acceptability of using AGDs[2] on elephants	Unacceptable	13.8	17.2	8.2	3.7	13.3	17.8
		Somewhat unacceptable	12.3	24.7	3.5	7.4	9.6	19.4
		Neutral	18.5	16.1	41.2	37.0	30.1	23.3
		Somewhat acceptable	38.5	26.9	31.8	40.7	36.1	26.7
		Acceptable	16.9	15.1	15.3	11.1	10.8	12.8
3. How effective do you think satellite linked shock collars would be in managing elephant movement?	Effectiveness of AGDs[2] in managing elephant movement	Ineffective	4.6	8.6	2.4	3.7	4.8	3.9
		Somewhat ineffective	9.2	17.2	7.1	0.0	8.4	2.3
		Neutral	20.0	21.5	29.4	25.9	33.7	26.7
		Somewhat effective	49.2	39.8	41.2	37.0	41.0	43.0
		Effective	16.9	12.9	20.0	33.3	12.0	24.0
4. If a pilot study using electric shock collars is conducted on captive elephants and is proven that elephants do learn to avoid shock when they hear the warning sound, would it then be acceptable to be tested on wild elephants	Acceptability, if pilot studies on captive elephants have been successful	Unacceptable	7.7	16.1	7.1	0.0	4.8	10.1
		Somewhat unacceptable	6.2	14.0	0.0	11.1	9.6	5.0
		Neutral	21.5	12.9	32.9	11.1	24.1	20.5
		Somewhat acceptable	33.8	31.2	18.8	22.2	28.9	34.5
		Acceptable	30.8	25.8	41.2	55.6	32.5	29.8

[1] HEC: human-elephant conflict

[2] AGDs: aversive geofencing devices

Table S4. Selected list of reasons provided by respondents for unacceptability of using aversive geofencing devices on elephants

Theme	Examples
Unethical/ cruel/ harmful to elephants	"Cruelty to animals"
	"It's not a solution I can agree, there has to be a natural way!, culturally not acceptable"
	"No animal should harm during a research. It is against their will"
	"Elephants should be left alone without being harmed in the forest. It will endanger their normal lives"
	"I disagree with this concept as it would disturb the natural behaviour of elephants. It's no different than Jurassic Park T-Rex"
	"Cannot approve experimenting on animals to manage everything according to human needs"
	"This may violate natural habits of elephants and their senses"
	"They should be allowed to live their natural life"
	"Male elephant is usually more likely to go closer to human settlements and farm. The shock from the collar is something that the elephant cannot control unlike the electric fencing. I am afraid the shock will add to stress level of this elephant especially if the male elephant is in musth etc"
	"Unethical, inhumane and harmful. Such intrusive techniques on endangered species is completely unethical. Firstly this cruel technique would not be able to predict how the elephant will respond. It might make the animal more dangerous and uncontrollable."
	"Adding tracking collars alone affects the natural behaviour of the elephants. Having a shock collar may be effective but it may effect how they behave in the wild. Even when the shock isn't present, the sensation of the collar may cause fear and paranoia. In particular when this is put on the matriarch, it could affect the stability of the entire herd."
	"It may cause cardiac arrest or any other health related issues for the elephant"
	"Seems too cruel and controlling"
	"I am ethically against doing this to a wild animal."
	"This cruel and unethical. There are no problem elephants only problem locations. So site specific mitigation measures should be practiced to reduce/manage the human-elephant conflict. Just because there is a technology available and someone think this is novel and there is money to do this we shouldn't be doing do such tests/research."
	"Elephants should be able to live without human influence even without a collar. I will also affect the aesthetic beauty"
	"GPS electric collars are different from physical barriers such as electric fences. They have the potential to cause psychological trauma to elephants by giving the elephant a shock from a device which is installed on its own body. It is like installing electric collars on the necks of human prisoners."
	"Either captive or wild, the elephant has to be anesthetized to place the collar. It is a risk to their life. Shocking will affect their natural behaviours."
	"Cannot approve harming animals"
	"Involving captive elephants is also harmful"
	"Shock collars are inhumane, elephants may not understand what happens and may panic and aggravate the conflict. Animal right groups should oppose this"
	"Wild elephants are not the reason for the beginning of this conflict. The reason is the acts of humans. So, punishing and experimenting with using wild elephants is unacceptable and it is unethical. Although they are animals, they have the blood and flesh that feel the pain. Therefore, experimenting with painful stuff is unacceptable. There should have practical solutions which are not painful for elephants as well as for humans. These artificial solutions cannot cure this problem."

Theme	Examples
	"Because the elephant would learn that the sound is a warning signal for the shock, and it would constantly be in state of stress/anxiety whether it would get a shock. I believe this is inhumane."
	"Shock collars can create fear, anxiety and aggression in animas towards people or other animals. They are natural animals so have to let be"
	"Electric shock could harm or cause death to the animal, the animals mental health could be affected and could act even more aggressively"
	"Electric shock could harm the elephants"
	"Giving an electric shock to any animal cannot be accepted"
	"Animals are innocent, and testing shocks on elephants can harm them,"
	"No animal should be used for experiment. Animals should be protected"
	"There is a possibility that the shock could harm elephants, die or even act aggressively in response to the shock"
Would be unfeasible or unsuccessful	"Difficult task"
	"After wearing the collar, the Elephant herd might reject the individual elephant in my opinion this method may show some progress but not the solution."
	"Behaviour of captive elephants and wild elephants are different"
	"Because it will ultimately don't work for the wild as well."
	"Pilot tests on captive elephants will be probably successful, such as electric fencing is working very well with captive for instance. But it does not prove that the system will be efficient for wild ones in search of highly energetic food. Wild elephants may probably learn at the beginning but, with time, they could also learn that electric shocks will are a bearable pain (10-15 sec) for accessing farmed food, such as elephants learnt to cope with electric fencing. Apart from these potential technical limitations, installing permanent collars on wild elephants is highly intrusive with non-zero risks when sedating the animal. I don't believe that segregating humans and animals is the way to promote co-existence - should be more about land planning, community-led solutions, etc."
	"Captive elephants behave differently from wild elephants"
	"Not effective. Domestic elephants are more fond with people. If they were used, the may be adversed."
	"Firstly, elephants have to be captured and fitted with collars. These collars will have to stay on the elephants permanently unlike other radio collars which can be removed after a certain time. Instead of satellite linked collars, one can use trip alarms which will warn the farmers. Trip alarm are much cheaper and does not involve the capture of elephants."
	"The behavioural patterns of captive elephant may differ from wild elephants. Wild elephants are more tolerant to pain that captured ones."
	"Captive elephants may not behave the same way as wild elephants<br>If electric shock is effective in stopping male elephants then electric fences should also work, but it is not the case at present.<br>High cost to put collars on many elephants, practical problems in sedating animals, risk to humans involved in it.<br>Early warning systems through sms or alarm sounds when elephants cross virtual fences would be great."
	"Elephant being an intelligent species can also remembers it as a bad memory and can also show retaliation unlike captive elephant on which you have experimented and which is also used to humans.<br>Shock collars are more problematic than fences."
	"Captive elephant behaviour is different"
	"Captive elephants are different from wild. The direction in which the elephant would move after receiving the shock in uncertain. When stressed out the elephant might not know in which direction it should move"

Table S5. Selected list of feedback received from respondents for potential challenges and other comments on aversive geofencing devices

Theme	Examples
(1) Stakeholder acceptability, support and awareness	"Will be difficult to implement with the government"
	"Culturally not acceptable"
	"Selecting a suitable community to implement this will be difficult, Will need a lot of effort to educate and create awareness among people regarding this process. It will be challenging to coordinate between govt authorities, local communities and other institutions to implement this."
	"Adoption of technology by the affected communities"
	"Political influence"
	"Educating the villagers., Stakeholders must be adequately educated and corporation from the govt must be assured."
	"The attitudes of wildlife and environmental activists"
	"Employing local workers to see program through, corrupt politicians, local people trying to profit through implementation programs"
	"People living in conflict areas may initially be reluctant to accept this solution. Most of them are asking for electric fences. "
	"Animal right activists may oppose"
	"Coming into an agreement with the majority, educating villagers regarding the technology"
	"Ethical, religious, and cultural issues when implementing"
	"Legal issues and consent of the host country"
	"If the health and welfare harms of attaching and using an electric shock collar are outweighed by the desired effectiveness and benefits, then the ethical questions become once of risk/benefit balance. If elephants are considered as having rights, for example in accordance of the five freedoms or as 'non-human persons', then you'd probably have to find another way."
	"Animal rights groups will oppose use of this technology"
	"The ethical consideration when implementing these methods, specially how to explain them to wildlife conservationist and general public who loves elephants. Will governments spend more money on such technology that require a lot of investment, considering the cause of the problem and the existing methods they are already aware of."
	"One of the key challenges is how the message is communicated to the public (efficacy, risks, costs, etc). There are a lot of animal welfare activists these days, which is not wrong, but they may not always be practical, or they may not fully understand the issues on the ground. Also, this technology may work well for tuskers, but I am not sure about family groups; ie; there are no clear matriarchs for family groups in Sabah, Malaysia, based on my observation."
	"Attitude of environmentalists/ animal welfare activists/ community towards elephants being subjected to electric shock" training or coordination required for officials/ those who would conduct the monitoring
	"Preventing abuse in its use by statutory agencies (following political directive)."
	"Educating the communities about this, coming to an agreement to implement this with the government and with the laws and regulation in the country will be difficult"
	"Government support, unwillingness to take risks"
	"Education, people need to be educated about this. It also needs to be ethical"
	"Government support"
	"I think the key challenge will be managing animal rights activists and welfare groups. If an international animal rights groups hears of this practice occurring, the proponents will most likely experience a high level of online abuse and attacks by westerners. While potentially an effective solution, the perceived welfare issues will be very difficult to justify to animal rights groups. Animal rights groups are already making elephant conservation very difficult, and electric collars will fuel their online abuse (and they will gain more donations). Sadly, this may perpetuate the further racism and stereotypes about locals not caring for elephants. For this reason, I don't think electric collars are a good idea to mitigate HEC."

Theme	Examples
	"Farmers and civilians who live in areas with elephant interactions should be well educated for the method to be more effective."<br>"Lack of awareness among people"<br>"Lack of knowledge about this in people in rural areas"<br>"Legal issues, animal welfare/rights NGO’s that are against any kind of invasive treatment"<br>"The government should be encouraged for this"<br>"It can be implemented successfully only if the operational explanation related to the above vibration belt is given to the farmers and beneficiaries"
(2) Safety and wellbeing of elephants	"Should use a shock type that does not harm the elephant"<br>"Does the satellite link collar endanger the life of the animal"<br>"Elephants could be harmed mentally and physically affecting their lifestyles. Should ensure animals safety"<br>"The only problem that i have with it is the doubt of safety of the electric collar on the elephant. If it were to malfunction it will hurt the animal."<br>"Would expect minimal harm on elephants"<br>"Long-term impact on an elephant's behaviour is critical from a conservation point of view."<br>"Should ensure that the collars do not interfere with elephants physical "<br>"Poachers may be able to identify animals with their collars"<br>"Possible harm to elephants due to radiation, its best to see if there are long terms harms to elephants"<br>"Elephants could be harmed if the shocks are received multiple times"<br>"The inherent risks of demobilising an elephant "<br>I think this is a very serious and potentially harmful intrusion into the elephant's bodily autonomy and could have a potential to drive them "mad" instead of providing a viable solution.<br>"Wellbeing of the elephants."<br>"If elephants crosses all VFs then the action taken should not be harmful to the animal<br>There could negative effects on the animal (chances of cancer) due to receiving electric shock for long periods"<br>"Should ensure that it does not harm the elephant. "<br>"May cause long term effects to the individual, which might not come to light if unnoticed or research lacks."<br>"Giving electric shocks might negatively affect them and might do some damages to their nervous system if they frequently subjected to this."<br>"It is not good for their mental health"<br>"Increased stress level of the problem animal, shocks might increase the anger of the animal"<br>"Harm to elephant during capture"<br>"There is a risk anaesthetising an elephant and you don't want to do that with a well-behaved elephant (especially females)."<br>"Negative impact on elephant behaviour?"<br>"Warning collars would be alright as long as there's no physical harm to the animal."<br>"Ensuring that the pain experienced by the elephant is no greater than that delivered by an electric fence i.e. that there is no harm caused to the overall health and life of the animal."<br>"A safe way needs to be found for placing the collars. Using tranquilisers can be very dangerous for elephants and a full risk assessment and method statement needs to be prepared"<br>"Could it be disadvantageous to an elephant when involved in a fight, Poachers will be able to identify animals with the collars,"<br>"I hope this would not impact elephants in a negative way"<br>"Mental and physical health of elephants"

Theme	Examples
	"If a shock/set of shocks is missed when an elephant leaves a designated habitat area, there might be a risk of it getting shocks when it tries to re-enter the habitat area, leading to a traumatic and painful (and counterproductive) experience; logistics and health risks of repeatedly recapturing elephants if the collars need repair/replacement; Collaring younger animals that are still growing might risk strangulation, meaning collaring them more than occasionally could lead to a high monitoring burden/risk of unconscionable animal welfare costs"
	"Should find out if elephants mental health could be affected by this"
	"Could be life threatening to elephants"
	"Safety of animals"
	"Risk to animals during tranquilisation"
	"Mental stress to the animal"
	"Hope the electric shock from the collar is not life threatening to the elephants."
	" Will this shock affect the elephant's body, health and well-being of life in the long term? Is it harmful to their nerves, brain or any organ, Is it harmful to their population behaviour (mating, selecting a mating partner, competition) Is it changing their ecological behaviour? migration?"
	"Could interfere with elephants' natural behaviour"
	"Understanding the negative impact that the collar can cause to the animal"
	"Possible health problems that may arise as a result of wearing the collar for a long time. Collars should be developed in such a way that it won't cause any health problems to elephants"
(3) Logistical difficulties	"Economically demanding, logistically difficult to conduct."
	"Difficult task"
	"Initial cost"
	"High cost"
	"It will be very expensive, fixing collars also to animals is also very tough task."
	"How do we identify elephants in the wild that usually go to attack villages to put the collars? How to capture the wild elephants to wear the collars on their neck?"
	"High cost, difficulty in fitting them on elephants"
	"Trying to capture wild elephants"
	"To capture & fix the satellite linked collar on the wild elephants."
	"If a mobile system is included the availability on the phones and rated resources could be scarce in the remote areas where the issue is persistent."
	"To get proper technical knowledge, proper staffing for elephant handling"
	"We can't use collars for every elephant in the heard. Its big challenge for deciding and select Elephant to put a collar."
	"This can be only used on a number of elephants, so figuring out which ones to be used will be a problem."
	"All the farmers here do not have mobile phones like other countries "
	"Elephant behaviours are unique to each area and therefore each area and elephant behaviour should be studies before this can be implemented."
	"Tranquilizing and fixing the shock collars.<br>Monitoring 365 X 24 Hrs."
	"Will require lot of effort, many people and a lot of time"
	"Identification of key problem animals"
	"It will be difficult to put collar for large number of elephants and it would be a costly process"
	"It would be cost-prohibitive to use this on a scale that matters for managing elephants. cannot be deployed on hundreds of individuals over their lifetimes."

Theme	Examples
	"The challenge is to determine the exact location of a virtual electrical fence in a landscape that is completely fragmented. In addition, elephants often need to cross people's crops to move from one block of protected forest to the next. If this path is cut off with a virtual electrical fence, the conflicts will escalate. The underlying issue is not what type of electrical fence could be used, but to design an integrated elephant-friendly landscape."
	"Fixing the collars on all wild elephants"
	"Difficult task to fix collars on wild elephants"
	"Capturing wild elephants to install the collars would require a lot manpower."
	"Key challenge will be the implementation of the method which needs funding and training through workshops."
	"Extensive fieldwork and a dedicated team at the initial stage. Finding the problem elephants."
	"Applying the collars on elephants is certainly difficult/ dangerous"
	"The usual problems of capture and immobilization to fit a collar will exist and the responses of animals in the vicinity may also affect further collaring efforts. Until you start, it is difficult to determine how many elephants you would need to collar to make a consistent impact and the number could be large, if not all."
	"Scaling this up to cover large herds of elephants will be a challenge. For individual elephants, this would be somewhat manageable. cost of maintenance of such a system would be considerably high"
	"Fitting the collars on wild elephants. Even if it is done only on the matriarch, tranquilizing and capturing the matriarch to fit the collars might lead to anti-human feeling in the herd and they might want to harm humans in general."
	"Defining boundaries for virtual fences without severely compromising land available for elephants would be a key challenge especially in areas with no land-use plan. Deciding which individuals and how many individuals from a group should be fitted with a collar to effectively mitigate the conflict would be challenging."
	"Elephant living in a group and you cannot collar all of them but only few. The cost to collar elephant is expensive. The habitat need to be improve with proper land use planning, if they don't have enough room to live then this technique would certainly will not be effective. Combination with all the other mitigation strategies should also be applied."
	"Practicality in implementing in large areas with HEC"
	"Aside from the animal rights issues, there is the problem of installing electric collars on so many animals. Even if one decides to install the collars only on matriarchs, that is still a large number of animals that needs to be collared."
	"Expense of doing it at scale, including all the individuals/the right individuals (that might be leaders) in a group that participate in conflict; Collaring younger animals that are still growing might risk strangulation, meaning collaring them more than occasionally could lead to a high monitoring burden/risk of unconscionable animal welfare costs; Once more than a few elephants are collared, the government might lack the capacity necessary to properly track them and intervene."
	"I think the biggest challenge would be setting criteria of when it would be appropriate to use shock collars, and criteria of when it would be inappropriate to use shock collars. There will definitely be an element of trial and error, but there still be a robust justification of why to conduct the pilot study in a particular location/why you think the pilot study will be most likely to succeed in the location you choose."
	"Most wildlife departments and community organizations do not have the resources or technical capacity to do this."
	"High initial cost, Risk in fixing collars, lack of trained personnel, not enough people with experience to implement"
	"Identifying the problem elephants would be the primary issue for this approach. We are seeing in our tracking data that their movement behaviour are very diverse and identifying problem elephants can be challenging than previously thought since the elephants' resource use and strategy can change year-by-year."

Theme	Examples
	"Coming up with adequate funding for collars and implementation"
	"Rural communities may not have smart phones to receive information"
	"Financial issues, people with specialist technical knowledge"
	"High cost and will require trained people"
(4) Durability and reliable functionality of AGDs	"Prevent damage to the collars in the long-term wear."
	"Long term sustainability of the device, repairing the device, signal errors"
	"Elephants fast learners and intelligent, they will figure out a way to get the collars removed from their necks."
	"Elephants can damage the collar"
	"Technical issues with the device"
	"Network coverage in rural areas (hoping this system would need internet to communicate)"
	"Collars being broken or defects in collars can affect the results"
	"Ensuring all elephants have continuously working collars."
	"Keeping the collars charged. Weather interferences. Updating collars with elephant growth."
	"Natural behaviours/weather/bad maintenance can damage the belt."
	"Replacing batteries,"
	"Elephants might damage it's collar with time."
	"What about the durability of this collar? This will be using a power source like a battery to give an electric shock and sound alarms. So how long will this last? Most of the time troublesome ones are young male elephants, and they tend to fight each other very often. Can that be a problem? Can the collars be damaged and stop working? "
	"Managing and maintained of the collars, Satellite signals will be disrupted by dense forest, cloudy weather and other factors "
	"Poor satellite connection and signal issues"
	"Due to GPS signal loses in rural areas people will get false information about elephants"
	"Collar can be damaged when the elephant goes through the trees and bushes."
	"Should be protected from water."
	"Durability of the collars. Elephants will take off the collar."
	"They'll get used to it"
	"Damage to collar during fights, etc"
	"Battery life "
	"Collars have a battery life, require maintenance (periodic re-collaring), are prone to dysfunction, "
	"Durability and duration. Satellite collars are known to fail often. And the batteries have a life. How would you address these issues?"
	"Chances of elephants removing collars,"
	"Potential malfunctioning of the technology leaving elephants being shocked permanently or inappropriately - both of which might backfire. Elephants are VERY tough on technology, build it very strong & make sure it is fool proof."
	"The collars may malfunction and provide shock at other times or provide a shock of higher intensity than intended.<br>what will happen when the batteries on the collars expire?"
	"Elephants are smart and would certainly find the collar as source of pain and try to destroy it"
	"Durability of the collar, battery life, elephant behaviour could harm the collar, Elephant might find the shock troublesome and try to break the collar"
	"Depending on terrain, canopy cover etc., sufficient GPS linkage might be problematic. Elephants have tough loose skin, so contact points for electric collar might not make consistent connections, all of which might prevent or undo any learned behaviours."

Theme	Examples
	"Sometimes they might be able to remove the collars somehow by damaging them, If signalling systems are damaged or satellite signals or phone signals are low in some areas, then there might be some issues with making people aware about trespassing elephants. "
	"Limited lifetime of collar"
	"I am afraid of collar malfunction, Elephants are clever and will definitely link the electric shocks with the presence of the collar, thus leading to the elephants trying to remove the collar. This will be done in a brute force way with the elephant maybe rubbing it against a tree. This whole process introduces the possibility of malfunction and raises the probability that the elephant will either experience constant noise or constant electrical shocks from the collar. To remove the collar will then be another large stressful event for the elephant as it needs sedating. The possibility of failure with the collar worries me the most."
	"Battery life. the frequency discharging the electric shock and for how long it would discharge shock if animal is not moving from danger zone?"
	"Also, the collar lasts only for a little while."
	"Regulation of the electric charge"
	"Collar failure/signal disable will make harmful to local inhabitants (local people will presume they are protected but these satellite signal disable will cause no warning to them resulting in causality, I think)"
	"Effectiveness of the GPS in tracking elephants in largely forested areas"
	"Durability of the collar – water resistance etc."
	"Risk of elephants receiving shocks continuously"
	"The continuity of proper functioning of the device without any technical failure/ life span of the device? "
	"How the collars are going to be retained by elephants"
	"The satellite collars do not last long. The life span of battery has been always challenging to us. If your collars can have long lasting battery life, then it would be a game changing intervention to minimize HEC."
	"Reliable technological functionality, especially in more remote areas, but in general maintaining satellite connectivity at the right times might be a challenge, Battery life could be an issue; Elephants might (?) recognize the collar is hurting them and try to damage/remove it"
	"In case collars gets removed or detached from elephants' body, elephants take shelters in areas having no satellite connectivity, Satellite linked warning collar may get damaged"
	"Weather resistant collars/ durability of collars"
	"Battery issues and technical issues of the collar"
	"Early warnings might not reach the people properly"
	"Developing a reliable collar"
	"Communication signals might not work in some areas"
	"Durability of the collar, accuracy of collars"
	"How to ensure that all collar work successfully"
	"How the collars may be affected by weather, Chance of collar removing"
	"Satellite connection may break down depending on weather conditions"
	"There is also a chance that the collars would get damaged or could get removed, rainwater could affect the electric components of the collar"
(5) Uncertainties in wild elephants' responses to AGDs	"Training wild elephants to respond to stimuli from the collars "
	"Herd might reject the collar wearing elephant. That's the major drawback because Elephant are socialized animals"
	"If the heard learn that only matriarch is susceptible for these shocks, they will soon adopt against it. Probably make the matriarch wait outside the fenced area while the heard enter without getting shocked?"
	"Feel that elephants could habituate to it"

Theme	Examples
	"They will find a way to adopt that shock "
	" Maybe the elephant will adapt to the electric shock and need to develop a system to increase or variable the electric shock where necessary. Behavioural changes due to frequent electric shock. As we know, many elephants' habitats and migrating routings are within the human areas."
	"Variable sensitivity to shock from elephant to elephant"
	"It is possible that some elephants will continue to raid crops and risk the shock, such as bull elephants (esp in musth). The shock could also cause aggressive behaviour so this also should be monitored so no one is in the vicinity and in danger."
	"Elephants are extremely smart animals and if you only tracked the matriarch it's likely this elephant may learn to avoid human-dominated landscapes, but this may not be the case for the rest of the group, who may still incur while the matriarch forages elsewhere."
	"However, collar will be attached to one elephant of the herds. I don't know how rest of the member will react to this."
	"Habituating to the shock collar, Chances of elephant panicking and acting aggressively in response to the shock,"
	"Terrifying of the elephants leading to human elephant conflict"
	"habituation"
	"Elephants are absolute genius. They quickly adopt or decode. We can only put on lone tuskers or matriarch. So other elephants of the herd can still cause sufficient damage."
	"Elephants are intelligent and somewhat unpredictable from our perspective as a different species. Individuals varying greatly in their behaviours and reactions ('personalities') and the consequence of receiving one or several electric shocks with only locational and auditory, but no visual, cues is really unpredictable. Captive animals may not offer much insight, so field tests are probably required from the start."
	"May aggravate the animal to cause more destruction."
	"Elephants can be acclimatized to the electric shock if they are frequently subjected to this, If the electric shock is given only to the leader of the herd or few, then the other members might enter the villages and do the damage. "
	"Over the long term it could have unpredictable behaviour results. Imagine a herd of elephants moving towards a border. The matriarch receives a shock and starts moving away. Would that behaviour challenge his/her ability to lead the herd? Would it lead to internal conflicts in the herd?"
	"Behavioural and thinking patterns difference between captured and wild elephants. "
	"Elephants are intelligent animals and the collar on the matriarch might lead to change in behaviour of the herd towards her. Also, once the collared elephant realizes that the shock is not really painful and bearing through it helps the herd in fetching food, the method might prove ineffective compared to electrical fencing wherein the entire herd gets the electrical shock."
	"Based on my experiences, most of wild elephant do tolerate things more than captive elephant, so the voltage that use for wild elephant maybe need to be adjusted. Also, I'm not sure whether wild elephant will show similar result to captive or not. Anyway, please also be careful, if the shock or noise from collar will stimuli the aggressive of the wild elephant."
	"It assumes that matriarch or habitual troublemakers will drive other elephants away from the danger. In reality this won't happen. The other non-collared animals will cause HEC, probably to a lesser extent? There is a possibility of HEC to be aggravated since electric fences are not existing, at least when pilot study is underway with proposed method."
	"The boundaries then might also have to have electric fences or something which the elephant takes as the reason of shock so he will keep avoiding it after the collar stops working"
	"Elephants could also learn to get around the barrier effect over time."
	"A word of caution in that elephants are vastly intelligent animals and may not be as acquiescent as cattle and sheep. The drive to find food and water currently means that some individuals, even mature females, will tolerate the pain of an electric fence to reach it."
	"Every Elephant can react in different way. Outcome cannot be generalized."

Theme	Examples
	"One will also cause disturbance in the social dynamics of the animals, because the other elephants will not understand why the collared matriarch is refusing to go into a certain area. They can't understand that the collared matriarch is receiving electric shocks from the collar."
	"How will the other elephants react to the elephant wearing the collar, Difference in how individual elephants will respond to it."
	"In case the elephant breaches the virtual fence, there should be a backup mechanism to mitigate the conflict which would arise then."
	"Elephants will eventually learn to circumvent it , they have done it every method used by humans"
	"There is a risk of elephant's behaviour being influenced/hampered by shocking from installed in her/his neck collars that even can provoke more damage to human lives/property,"
	"Some elephants (a minority) might get habituated"
	"The key challenges are the tremendous plasticity in elephant behaviour and their ability to adapt. As with other management techniques, I imagine that for some individual elephants the collars might work but perhaps not for all. Some elephants could potentially become more aggressive and go on a rampage after being shocked."
	"The time taken for elephants to learn the process"
	"Elephants could get aggressive as it disturbs their natural behaviour"
	"Since there is no physical barrier, if the elephant panics after the shock it might not be able to figure out the direction in which it should move"
	"If elephants walk forward ignoring the stimuli it will be difficult to be safe from the elephant"
	"This method might not be effective if the elephant gets used to the shock and sound"
	"Issues with sensitivity of elephants"
